# Brain abscess in Honduras: a five-year clinical and epidemiological study at Hospital Escuela

**DOI:** 10.3389/fneur.2026.1826171

**Published:** 2026-07-17

**Authors:** Jesús Rodríguez, Daniel Pineda, Melania Madrid, Marcio Madrid, Lilian Sosa, Lupe Carolina Espinoza, Isaac Zablah

**Affiliations:** 1 Faculty of Medical Sciences, National Autonomous University of Honduras, Tegucigalpa, Honduras; 2 Faculty of Biology, University of Salamanca, Salamanca, Spain; 3 Universidad Pedagógica Nacional Francisco Morazán, Tegucigalpa, Honduras; 4 Institute of Microbiological Research (IIM), Faculty of Sciences, National Autonomous University of Honduras (UNAH), Tegucigalpa, Honduras; 5 Department of Pharmaceutical Technology, Faculty of Chemical Sciences and Pharmacy, National Autonomous University of Honduras (UNAH), Tegucigalpa, Honduras; 6 Department of Chemistry, Faculty of Exact and Natural Sciences, Universidad Técnica Particular de Loja, Loja, Ecuador

**Keywords:** brain abscess, developing countries, epidemiology, Honduras, neuroinfections

## Abstract

**Background:**

Brain abscess is a focal, life-threatening infection of the central nervous system associated with high morbidity and mortality, remaining scarcely studied in low-income settings. This study describes the clinical and epidemiological profile of patients with brain abscess at Hospital Escuela, Honduras (2018–2023), including treatment patterns and in-hospital outcomes.

**Methods:**

A retrospective descriptive case series was conducted at a tertiary referral hospital. Thirteen medical records were identified through ICD-10 coding (G06.0–G06.2). With diagnoses confirmed by radiological, microbiological, or documented clinical-team criteria. Analyzed variables included demographics, predisposing conditions, neuroanatomical location, number and estimated volume of abscesses, neuroimaging modality, microbiological findings, empirical and pathogen-directed antibiotic regimens, intensive care admission, and modified Rankin Scale at discharge. Descriptive statistics (frequencies and proportions with 95% confidence intervals by the Wilson method) were used. The study followed the STROBE reporting guidelines.

**Results:**

Of 13 patients, 61.5% were male, and 53.8% were pediatric (≤15 years; range 8–14 years). All abscesses were supratentorial (100%; 95% CI: 77.0–100.0%), with parietal and occipital lobes each affected in 30.8% of cases. Traumatic brain injury was the most frequent individual predisposing condition (23.1%; all three cases pediatric). Etiologic identification at any level was achieved in 30.8% of cases; direct microbiological confirmation from cerebrospinal fluid was available in one case. Identified organisms included *Streptococcus* spp. and uncommon opportunistic pathogens (*Burkholderia pseudomallei, Cryptococcus neoformans, Staphylococcus* spp., and *Nocardia* spp.). Neurological status at admission was preserved in 84.6% (GCS 13–15). All patients were managed exclusively with intravenous antimicrobial therapy; no patient underwent neurosurgical drainage. In-hospital mortality was 0, and 46.2% of patients were discharged with no significant disability (mRS 0–1), 30.8% with slight disability (mRS 2), and 23.1% with moderate disability (mRS 3).

**Conclusion:**

This first institutional clinical-epidemiological characterization of brain abscess in Honduras demonstrates a pediatric-skewed cohort, a low etiologic identification rate, and a management pattern characterized by exclusive medical therapy. Survival was universal, but substantial neurological morbidity persisted at discharge. The findings should be interpreted as descriptive of a single national referral center and provide baseline data for future multicenter research in Central America.

## 1 Introduction

Brain abscess is defined as a focal accumulation of pus within the brain parenchyma, typically encapsulated in its mature phase and surrounded by a zone of cerebral edema that can cause significant neurological injury (1). Its clinical course ranges from subacute to fulminant presentations, and the classic triad of headache, fever, and focal neurological deficit is present in fewer than 60% of cases, accounting for the frequent diagnostic delays reported in the literature (2, 3).

Global epidemiology indicates a relatively low incidence in high-income countries, ranging between 0.3 and 0.9 cases per 100,000 inhabitants per year (4, 5). In resource-limited regions, however, incidence is likely higher, driven by adverse socioeconomic conditions, greater prevalence of untreated otorhinolaryngological infections, and restricted access to advanced neuroimaging (6, 7). The 2024 European Society of Clinical Microbiology and Infectious Diseases (ESCMID) guidelines estimate that brain abscesses account for approximately 8% of intracranial masses in low-resource settings, compared to 1–2% in high-resource environments (8).

The most common predisposing conditions include contiguous infections (otitis media, sinusitis, mastoiditis), hematogenous dissemination from distant foci such as endocarditis or pulmonary infections, and immunosuppressive states including diabetes mellitus, HIV infection, and long-term corticosteroid use (9, 10). A large systematic review and meta-analysis comprising 9,699 patients identified Streptococcus sp. and Staphylococcus sp. species as the most frequent causative microorganisms, accounting for 34 and 18% of positive cultures, respectively (11). Tropical and developing-country settings are also associated with distinctive etiologies, including free-living amoebae, *Burkholderia pseudomallei* (melioidosis), and *Mycobacterium tuberculosis* (12).

In Latin America, published data are scarce and derive predominantly from retrospective hospital series with limited sample sizes (13, 14). A recent Colombian study reported 43 cases, highlighting persistent challenges in microbiological diagnosis and high rates of neurological sequelae (15). Changes in epidemiological patterns have also been observed in the post-COVID-19 period, with pediatric brain abscess incidence increasing markedly in some Asian centers (16).

In Honduras, published evidence on brain abscess is limited, largely based on case reports. A brain abscess associated with neurocysticercosis has been described in an adult patient treated at the Hospital Escuela, who presented with headache, vomiting, and focal neurological deficits, confirmed by neuroimaging, and with favorable medical-surgical management. A brain abscess secondary to odontogenic infection was also reported in an adolescent from Olancho, characterized by fever, progressive neurological symptoms, and a space-occupying lesion in the temporal lobe that required surgical drainage. Historically, a case of *Salmonella Typhi* meningitis complicated by a brain abscess in an infant has also been documented, highlighting the etiological diversity of this pathology in the country and the scarcity of systematic clinical-epidemiological studies on this condition in the Honduran population (17–19).

Therefore, this study aimed to describe the clinical and epidemiological characteristics of patients diagnosed with brain abscesses treated at Hospital Escuela in Honduras between 2018 and 2023.

## 2 Materials and methods

### 2.1 Study design and setting

A retrospective descriptive case series was conducted at a tertiary referral hospital, following the STROBE (Strengthening the Reporting of Observational Studies in Epidemiology) guidelines (20). Hospital Escuela is the national tertiary-care referral center in Honduras, located in Tegucigalpa and serving a catchment population of approximately 9.5 million inhabitants (21). The study encompassed all medical records of patients with a confirmed brain abscess diagnosis between January 2018 and December 2023. See Supplementary File S2.

### 2.2 Case identification and diagnostic criteria

Cases were identified through the institutional discharge database using ICD-10 codes G06.0, G06.1, and G06.2 (22). Diagnosis of brain abscess was established by at least one of the following criteria, applied in conjunction with the discharge code: (i) radiological identification of a ring-enhancing intracranial lesion on CT or MRI in the appropriate clinical context; (ii) direct microbiological evidence consistent with brain abscess obtained from cerebrospinal fluid, blood culture, or serology, supported by clinical and radiological findings; or (iii) documented neurosurgical-team clinical assessment supported by laboratory or microbiological findings in patients for whom contemporary neuroimaging was not available. Cases identified solely through ICD-10 coding without confirmatory clinical, radiological, or microbiological documentation were excluded. The diagnostic criteria adopted in this study are broader than those used in series that require histopathological or intraoperative confirmation, reflecting the practical reality that no patient in this cohort underwent surgical sampling. The implications of this permissive case definition are addressed in the Limitations section. Conditions specifically excluded included subdural empyema, epidural abscess, postoperative sterile fluid collections, and ring-enhancing lesions subsequently reclassified as neoplastic.

The three cases without documented neuroimaging were retained because diagnosis was confirmed intraoperatively and/or by direct microbiological evidence documented in the medical record. No case was retained based on clinical judgment alone. Inclusion criteria required a confirmed diagnosis under the above criteria and sufficient clinical information to extract core variables. Records outside the study period or with insufficient data were excluded.

Competing diagnoses commonly encountered in tropical and resource-limited settings, including tuberculoma, neurocysticercosis, toxoplasmosis, and ring-enhancing neoplastic lesions, were addressed by clinical context, response to empirical antimicrobial therapy with serial radiological follow-up, and selective microbiological or serological workup when clinically warranted (for example, exclusion of mycobacterial and fungal etiologies in Case 10 by AFB smear, *Mycobacterium tuberculosis* PCR, and fungal serology). Systematic serological screening for neurocysticercosis and toxoplasmosis was not performed in all cases; the implications are addressed in the Limitations section.

### 2.3 Variables and data collection

Variables analyzed included: sex; age group (reported both as continuous variable and as age group: pediatric ≤15 years, adult 16–59 years, elderly ≥60 years); predisposing conditions; primary neuroanatomical location of the dominant lesion; number of abscesses (solitary vs. multiple); estimated abscess volume (<1, 1–10, or >10 cm^3^, calculated by the ABC/2 method from available neuroimaging reports); Glasgow Coma Scale (GCS) at admission; neuroimaging modality; and microbiological and etiologic findings. Treatment variables were also extracted and included neurosurgical intervention (yes/no, and modality if applicable), empirical antibiotic regimen on admission, pathogen-directed regimen if microbiological identification prompted a transition, total duration of intravenous antimicrobial therapy, oral step-down (yes/no), and admission to the intensive care unit. In-hospital outcome variables included survival to discharge, modified Rankin Scale (mRS) at discharge, and specific neurological sequelae (epilepsy requiring chronic antiepileptic therapy, visual field deficit, cognitive impairment, hearing loss, motor deficit). Predisposing conditions were classified as infectious (meningitis), metabolic (diabetes mellitus), traumatic (TBI), neoplastic, surgical, congenital, or toxicological. Data was extracted using a standardized, pre-tested instrument. A data harmonization stage was implemented to standardize categorical variables before analysis.

One record (Case 11, pediatric age group) contained an entry of “alcohol use disorder” as a predisposing condition. Given the clinical implausibility in a pediatric patient, this was retained in descriptive tables with an explanatory footnote but interpreted cautiously. Verification against the primary medical record is recommended.

### 2.4 Statistical analysis

Given the descriptive and exploratory nature of this case series (n = 13), analysis was limited to frequencies, proportions, and 95% confidence intervals (CIs) calculated using the Wilson method, which provides more accurate interval estimates than the Wald approximation for small samples (23, 24). No formal hypothesis tests were conducted, as the sample size is insufficient to support meaningful inferential conclusions. All estimates are presented with their 95% CIs to convey the degree of statistical imprecision inherent in a small-sample descriptive study. Complete statistical calculations and methodological justification are provided in Supplementary Material S1. Analyses were performed using SPSS version 26.0 and Python (25, 26).

### 2.5 Ethical considerations

The study was conducted in accordance with the Declaration of Helsinki (27). Ethical approval was obtained from the Biomedical Research Ethics Committee (CEIB) of the Faculty of Medical Sciences, National Autonomous University of Honduras (protocol number 072–2023), with authorization to access anonymized medical records.

## 3 Results

### 3.1 Demographic characteristics

Thirteen patients with a confirmed brain abscess diagnosis were identified during the study period. In this institutional series, 61.5% of cases occurred in males (8/13; 95% CI: 35.9–81.0%) and 38.5% in females (5/13). The pediatric age group (≤15 years) represented 53.8% of cases (7/13; 95% CI: 28.9–76.8%). Adults aged 16–59 years accounted for 15.4% (2/13; 95% CI: 4.3–42.2%), while patients aged ≥60 years constituted 30.8% of the cohort (4/13; 95% CI: 12.7–57.6%). The wide confidence intervals reflect the limited sample size and preclude formal inferential conclusions (Table 1; see also Supplementary Figure S1 and Supplementary Table S2).

**Table 1:** Demographic characteristics of patients with brain abscess (*n* = 13).

Variable	*n* (%)	95% CI (Wilson)
Male sex	8 (61.5)	35.9–81.0%
Female sex	5 (38.5)	19.0–64.1%
Age ≤15 years (pediatric)	7 (53.8)	28.9–76.8%
Age 16–59 years (adult)	2 (15.4)	4.3–42.2%
Age ≥60 years (elderly)	4 (30.8)	12.7–57.6%

CI, confidence interval. 95% CI by the Wilson method.

Within the pediatric subgroup (*n* = 7), individual ages were 8, 9, 11, 12, and 14 years, with three patients aged 14 years; no cases occurred in infants or preschool-age children. The adult subgroup (*n* = 2) comprised patients aged 33 and 48 years, and the elderly subgroup (*n* = 4) ranged from 62 to 67 years. Per-patient demographic data are reported in Supplementary Table S1.

### 3.2 Predisposing conditions

In this series, predisposing conditions were heterogeneous, with no single category clearly dominant (Table 2; see also Supplementary Figure S3 and Supplementary Table S8). Traumatic brain injury (TBI) was the most frequent individual predisposing condition, identified in 23.1% of cases (3/13; 95% CI: 8.2–50.3%). Diabetes mellitus and meningitis were each documented in 15.4% of cases (2/13; 95% CI: 4.3–42.2%). Less frequent conditions included cancer, prior neurosurgical intervention, and congenital hydrocephalus (each 7.7%; 95% CI: 1.4–33.3%). Two patients (15.4%) had no identifiable predisposing condition. One additional record contained an entry of alcohol use disorder in a pediatric patient (Case 11), which could not be verified against the primary medical record before submission and was therefore excluded from the main analysis; it is retained in the complete patient-level dataset (Supplementary Table S1) with an explanatory footnote. Overall, conditions classifiable as infectious (meningitis) were identified in 15.4% of cases, while traumatic, metabolic, and other non-infectious predisposing factors accounted for the remainder.

**Table 2:** Predisposing conditions (*n* = 13).

Predisposing condition	Category	*n* (%)	95% CI (Wilson)
Traumatic brain injury (TBI)	Traumatic	3 (23.1)	8.2–50.3%
Diabetes mellitus	Metabolic	2 (15.4)	4.3–42.2%
Meningitis	Infectious	2 (15.4)	4.3–42.2%
Cancer (immunocompromised)	Neoplastic	1 (7.7)	1.4–33.3%
Previous neurosurgical intervention	Surgical	1 (7.7)	1.4–33.3%
Congenital hydrocephalus	Congenital	1 (7.7)	1.4–33.3%
Alcohol use disorder †	Toxicological	1 (7.7)	1.4–33.3%
None identified	—	2 (15.4)	4.3–42.2%

†Documented in a pediatric patient (Case 11); probable data entry error requiring primary record verification. Multiple predisposing conditions were permitted per patient. TBI, traumatic brain injury; CI, confidence interval.

### 3.3 Neuroanatomical location

All 13 cases involved supratentorial locations (100%; 95% CI: 77.0–100.0%), encompassing frontal, parietal, temporal, and occipital lobes, as well as basal ganglia. No case involved infratentorial structures. Using the dominant lesion as the pre-specified classification rule, parietal and occipital lobes were each involved in 30.8% of cases (4/13), followed by the frontal lobe in 23.1% (3/13), and the temporal lobe and basal ganglia in 7.7% each (1/13) (Table 3; see also Supplementary Figure S2 and Supplementary Table S5). The combined parieto-occipital territory represented the dominant anatomical territory in this series (61.5%; 95% CI: 35.9–81.0%).

**Table 3:** Neuroanatomical location of brain abscesses (dominant lesion site; *n* = 13).

Location*	*n* (%)	95% CI (Wilson)
*Supratentorial (total)*	13 (100.0)	77.0–100.0%
Parietal lobe	4 (30.8)	12.7–57.6%
Occipital lobe	4 (30.8)	12.7–57.6%
Frontal lobe	3 (23.1)	8.2–50.3%
Temporal lobe	1 (7.7)	1.4–33.3%
Basal ganglia	1 (7.7)	1.4–33.3%
Infratentorial	0 (0.0)	0.0–23.0%

*Location reflects the dominant lesion site per patient. One case involved two anatomical regions simultaneously; the larger lesion determined primary classification. CI, confidence interval.

### 3.4 Clinical presentation

The most frequent presenting symptoms were convulsions and headache, each documented in 46.2% of cases (6/13; 95% CI: 23.7–70.6%), followed by fever in 38.5% (5/13; 95% CI: 19.0–64.1%). Less common manifestations included purulent discharge (15.4%), focal neurological deficit (7.7%), ocular protrusion (7.7%), and blurred vision (7.7%). The classic triad of headache, fever, and focal neurological deficit was documented in at most two patients, consistent with established literature reporting its low frequency (2, 8) (Table 4; see also Supplementary Figure S3B and Supplementary Table S3).

**Table 4:** Clinical manifestations and GCS at admission (*n* = 13).

Clinical finding	*n* (%)	95% CI (Wilson)
Convulsions	6 (46.2)	23.7–70.6%
Headache	6 (46.2)	23.7–70.6%
Fever	5 (38.5)	19.0–64.1%
Purulent discharge	2 (15.4)	4.3–42.2%
Focal neurological deficit	1 (7.7)	1.4–33.3%
Ocular protrusion	1 (7.7)	1.4–33.3%
Blurred vision	1 (7.7)	1.4–33.3%
GCS 13–15 (mild impairment)	11 (84.6)	57.8–95.7%
GCS 9–12 (moderate impairment)	2 (15.4)	4.3–42.2%
GCS 3–8 (severe impairment)	0 (0.0)	0.0–23.0%

GCS, Glasgow coma scale. Multiple symptoms were permitted per patient.

Neurological status at admission was preserved in the majority: 84.6% of patients (11/13; 95% CI: 57.8–95.7%) had a GCS of 13–15 (mild impairment), and 15.4% (2/13) had a GCS of 9–12 (moderate impairment). No patient presented with severe neurological impairment (GCS 3–8).

### 3.5 Abscess characteristics and neuroimaging

Solitary abscesses were documented in 61.5% of cases (8/13; 95% CI: 35.9–81.0%) and multiple abscesses in 38.5% (5/13). Most abscesses were medium-sized (1–10 cm^3^: 69.2%, 9/13), estimated by the ABC/2 method from available neuroimaging reports. Small abscesses (<1 cm^3^) were observed in 23.1% (3/13) and large abscesses (>10 cm^3^) in 7.7% (1/13). Full abscess characteristics are shown in Table 5 (see also Supplementary Figures S4A,B).

**Table 5:** Abscess characteristics and neuroimaging (*n* = 13).

Characteristic	*n* (%)	95% CI (Wilson)
Solitary abscess	8 (61.5)	35.9–81.0%
Multiple abscesses	5 (38.5)	19.0–64.1%
Volume <1 cm^3^	3 (23.1)	8.2–50.3%
Volume 1–10 cm^3^	9 (69.2)	42.4–87.3%
Volume >10 cm^3^	1 (7.7)	1.4–33.3%
CT scan only	5 (38.5)	19.0–64.1%
MRI only	4 (30.8)	12.7–57.6%
CT and MRI	1 (7.7)	1.4–33.3%
No imaging documented ‡	3 (23.1)	8.2–50.3%

‡ Confirmed by intraoperative and/or direct microbiological evidence. CT, computed tomography; MRI, magnetic resonance imaging; CI, confidence interval.

Computed tomography (CT) was the most frequently employed neuroimaging modality (38.5%, 5/13), followed by MRI (30.8%, 4/13) and both modalities in one patient (7.7%). Three cases (23.1%) had no documented neuroimaging; these were confirmed by intraoperative and/or direct microbiological evidence (see Supplementary Table S5 for neuroimaging details).

Considered jointly with the diagnostic criteria defined in the Methods, 10 of 13 cases (76.9%) were identified by ring-enhancing lesions on CT or MRI in the appropriate clinical context; 1 of these (Case 1) was additionally supported by direct microbiological evidence from cerebrospinal fluid. The remaining 3 cases (23.1%; Cases 5, 8, and 13) were attributed without contemporary neuroimaging, by documented neurosurgical-team clinical assessment supported by cerebrospinal fluid or serological findings. No case was confirmed by intraoperative or histopathological brain tissue analysis (Supplementary Table S5).

### 3.6 Laboratory and microbiological findings

Leukocytosis was documented in 69.2% of cases (9/13; 95% CI: 42.4–87.3%). Cerebrospinal fluid analysis, when performed, predominantly showed hyperproteinorrhachia; lumbar puncture was not universally conducted owing to concerns about elevated intracranial pressure. C-reactive protein was sporadically documented and was not uniformly measured, precluding systematic analysis (see Supplementary Table S4).

Etiologic attribution of any level was achieved in 30.8% of cases (4/13; 95% CI: 12.7–57.6%), whereas direct microbiological confirmation through cerebrospinal fluid culture and staining was available in only one case (Case 1); 69.2% of cases remained without etiologic identification. Three distinct levels of microbiological evidence were documented: (i) culture-confirmed: one case with cerebrospinal fluid culture combined with cytological and microbiological staining (India ink for Cryptococcus, Gram for Staphylococcus, modified Ziehl-Neelsen for Nocardia); (ii)presumed on the basis of clinical syndrome with supporting cerebrospinal fluid findings or serology: two cases; and (iii) indirect or unspecified: one case. No brain tissue was obtained from any patient, as no surgical intervention was performed during the index hospitalization (see “Treatment Approach and Clinical Outcomes” below). Full microbiological details are provided in Table 6 (see also Supplementary Figure S4C and Supplementary Table S6).

**Table 6:** Microbiological and presumed etiologic findings in identified cases (*n* = 4).

Case	Pathogen(s)	Evidence category	Clinical context
1	*Cryptococcus neoformans*/Staphylococcus spp./Nocardia spp.	Culture-confirmed (CSF) + microbiological staining	Severely immunocompromised patient with cancer. Etiologic attribution based on combined microbiological evidence obtained from cerebrospinal fluid analysis; not all organisms were recovered through the same laboratory modality. Cultures, India ink preparation, Gram stain, and modified Ziehl-Neelsen staining were performed on the same cerebrospinal fluid specimen. No brain tissue was obtained, as the deep basal ganglia location and the patient’s severe immunocompromise contraindicated surgical sampling.
5	Streptococcus spp.	Serology/CSF-supported (presumed)	Diabetic patient with prior neurosurgical intervention.
8	Unspecified organisms	Indirect (CSF findings only)	Secondary to bacterial meningitis; no direct culture of brain tissue.
13	*Burkholderia pseudomallei*	Serology-supported (presumed)	Elderly patient; melioidosis in a tropical context. *B. pseudomallei* is the current taxonomic designation (formerly *Pseudomonas pseudomallei*).

CSF, cerebrospinal fluid. Culture-negative cases (*n* = 9; Cases 2, 3, 4, 6, 7, 9, 10, 11, 12) are detailed in Table S6.

### 3.7 Treatment approach and clinical outcomes

All 13 patients were managed exclusively with intravenous antimicrobial therapy and supportive care. No patient underwent therapeutic neurosurgical drainage, craniotomy, or stereotactic aspiration during the index hospitalization (0/13; 95% CI: 0.0–23.0%). The decision against surgical intervention reflected case-specific factors that included deep-seated lesion with concurrent severe immunocompromise (Case 1), small abscess volume below 1 cm^3^ amenable to medical management (Cases 4, 7, 11), multiple bilateral lesions that limited a meaningful surgical target (Case 10), preserved neurological status permitting conservative management (Cases 2, 6, 13), and predisposing comorbidities that dominated clinical priorities (Cases 3, 5, 9, 12). Institutional factors, including the routine availability of stereotactic neurosurgical capacity and intraoperative neuroimaging during the study period, also contributed to the conservative pattern observed.

Empirical antimicrobial regimens followed a three-tier pattern (Table 7). A ceftriaxone–metronidazole backbone was used in 5 of 13 patients (38.5%; 95% CI: 19.0–64.1%) with solitary abscesses and no hardware, wound, or immunosuppression (Cases 2, 4, 7, 9, 13). Vancomycin was added to the backbone in another 5 patients (38.5%; 95% CI: 19.0–64.1%) when methicillin-resistant *Staphylococcus aureus* coverage was indicated by shunt presence, prior neurosurgery, post-traumatic mechanism, or purulent wound discharge (Cases 3, 5, 6, 11, 12). A meropenem-based broad-spectrum regimen was used in 3 patients (23.1%; 95% CI: 8.2–50.3%) with severe immunocompromise or meningitis-complicated multiple abscesses (Cases 1, 8, 10). Two patients required transition to pathogen-directed therapy: Case 1, with confirmed polymicrobial cryptococcal, staphylococcal, and nocardial infection, received combined antifungal, antinocardial, and antistaphylococcal therapy planned for 6 to 12 months; Case 13, with presumed central nervous system melioidosis (*Burkholderia pseudomallei*), received intensive intravenous meropenem followed by prolonged oral trimethoprim–sulfamethoxazole eradication. The duration of intravenous antimicrobial therapy ranged from 4 to 12 weeks (modal 6–8 weeks). Oral step-down to amoxicillin–clavulanate plus metronidazole was implemented in 4 patients (30.8%; 95% CI: 12.7–57.6%) after documented clinical and radiological improvement.

**Table 7:** Treatment approach and in-hospital outcomes (*n* = 13).

Variable	Category	*n* (%)	95% CI (Wilson)
Neurosurgical intervention	None	13 (100)	77.0–100.0%
	Drainage/aspiration/craniotomy	0 (0)	0.0–23.0%
Empirical regime	Ceftriaxone + metronidazole (backbone)	5 (38.5)	19.0–64.1%
	Backbone + vancomycin	5 (38.5)	19.0–64.1%
	Meropenem-based broad-spectrum	3 (23.1)	8.2–50.3%
Pathogen-directed transition	Yes	2 (15.4)	4.3–42.2%
	No	11 (84.6)	57.8–95.7%
IV antibiotic duration	<6 weeks	1 (7.7)	1.4–33.3%
	6–8 weeks	8 (61.5)	35.9–81.0%
	>8 weeks	4 (30.8)	12.7–57.6%
Oral step-down	Implemented	4 (30.8)	12.7–57.6%
	Not implemented	9 (69.2)	42.4–87.3%
ICU admission	Yes	5 (38.5)	19.0–64.1%
	No	8 (61.5)	35.9–81.0%
In-hospital mortality	Death	0 (0)	0.0–23.0%
	Survival to discharge	13 (100)	77.0–100.0%
mRS at discharge	0–1 (no/minor disability)	6 (46.2)	23.7–70.6%
	2 (slight disability)	4 (30.8)	12.7–57.6%
	3 (moderate disability)	3 (23.1)	8.2–50.3%
	4–6 (severe disability or death)	0 (0)	0.0–23.0%
Specific sequelae	Long-term epilepsy	6 (46.2)	23.7–70.6%
	Homonymous hemianopia	2 (15.4)	4.3–42.2%
	Cognitive impairment	2 (15.4)	4.3–42.2%
	Sensorineural hearing loss	1 (7.7)	1.4–33.3%
	Residual hemiparesis	1 (7.7)	1.4–33.3%

CI, confidence interval; ICU, intensive care unit; IV, intravenous; mRS, modified rankin scale. Multiple sequelae permitted per patient. Detailed per-patient antimicrobial regimens, durations, and clinical evolution are presented in Supplementary Table S1. Estimates are presented as *n* (%) with 95% confidence intervals by the Wilson method.

Five patients (38.5%; 95% CI: 19.0–64.1%) required admission to the intensive care unit, primarily for depressed Glasgow Coma Scale at admission or high abscess burden (Cases 1, 3, 8, 9, 10). In-hospital mortality was 0 of 13 (0%; 95% CI: 0.0–23.0%); all patients survived to discharge. Functional status at discharge, assessed by the modified Rankin Scale, was 0–1 (no significant disability) in 6 patients (46.2%; 95% CI: 23.7–70.6%; Cases 2, 4, 6, 7, 11, 13), 2 (slight disability, independent) in 4 patients (30.8%; 95% CI: 12.7–57.6%; Cases 3, 5, 10, 12), and 3 (moderate disability, partially dependent) in 3 patients (23.1%; 95% CI: 8.2–50.3%; Cases 1, 8, 9). No patient was discharged with severe disability (mRS 4–5). Documented neurological sequelae included long-term epilepsy requiring chronic antiepileptic therapy in 6 patients (46.2%), homonymous hemianopia attributed to occipital cortex involvement in 2 patients (15.4%; Cases 9 and 12), cognitive impairment in 2 patients (15.4%; Cases 8 and 10), bilateral sensorineural hearing loss attributed to antecedent meningitis in 1 patient (7.7%; Case 8), and mild residual contralateral hemiparesis in 1 patient (7.7%; Case 1).

## 4 Discussion

### 4.1 Demographic patterns

In this institutional series, 61.5% of cases occurred in males, a distribution consistent with the modest male predominance reported in large international registries (11, 28). Brandt and Bodilsen, in a nationwide Danish cohort of 1,384 patients over 35 years, reported approximately 37% female representation (29). However, the wide confidence interval in our series (35.9–81.0%) reflects the inherent imprecision of small-sample estimates, and no conclusions regarding sex distribution can be generalized from these data.

The pediatric representation in this institutional series (53.8%) is higher than that in published series from high-income settings, where pediatric cases typically account for 15–30% of brain abscess cohorts (2, 30). For regional comparison, the contemporary Colombian series by Acosta Pérez et al. reported a pediatric proportion of 23.3% (*n* = 43, high-complexity university hospital) (15), an intermediate figure consistent with high-income reports but below the proportion observed in our cohort; a full descriptive comparison with selected international series is presented in Supplementary Table S7. Within the pediatric subgroup, all patients fell between 8 and 14 years of age, with no cases in infants or preschool-age children. This concentration in school-age and adolescent patients departs from the broader age range described in pediatric reviews and is consistent with the predominance of post-traumatic and post-infectious causes in this stratum (31). The observed bimodal distribution, with concurrent contributions from school-age children and elderly patients, differs from the distribution in high-income countries, where the third decade of life predominates (8). Published reviews have documented a gradual decline in pediatric brain abscess incidence and mortality in recent decades, attributable to improved antibiotic therapy, refinements in neurosurgical technique, and expanded vaccination coverage (31). Several factors plausibly contributed to the pediatric over-representation in our cohort. Hospital Escuela functions as a national referral center for complex pediatric pathology and is the primary tertiary facility for children with severe traumatic and infectious neurological conditions in the country, which produces a referral skew toward pediatric cases. The proportion observed should therefore be interpreted as a feature of the institutional case mix rather than as an estimate of population-level age distribution in Honduras.

### 4.2 Supratentorial location

The supratentorial location of all cases in this series is consistent with the pattern reported in the international literature, where more than 80%–90% of brain abscesses occur in supratentorial regions (32). The 2024 ESCMID guidelines confirm this distribution (8). The predominance of parietal and occipital involvement (61.5% combined) differs from the frontal lobe predominance typically described in larger series (33). However, this observation should be interpreted cautiously, given the small sample size and the inherent referral bias in a single tertiary-care center.

### 4.3 Predisposing conditions and Etiology

Predisposing conditions in this cohort were heterogeneous. Traumatic brain injury accounted for 3 of 13 cases (23.1%); notably, all three patients were pediatric, aged 8, 11, and 14 years. The clinical implication of this concentration is uncertain in a series of this size. Several explanations are plausible and not mutually exclusive: referral and ascertainment bias toward a tertiary pediatric trauma service at Hospital Escuela; diagnostic bias arising from the permissive case definition adopted in this study (Methods, Case Identification and Diagnostic Criteria); and a true epidemiological contribution from elevated rates of pediatric trauma in Honduras, where road traffic injuries and violence-related trauma affect children disproportionately (34). A nationwide Danish case–control study identified neurosurgical procedures, cancer, and otorhinolaryngological infections as the leading risk factors for adult brain abscess (29). The traumatic profile observed at our center departs from that pattern, but the present series cannot disentangle the contributions of selection, ascertainment, and true epidemiological signal. These findings should not be extrapolated to other Honduran centers or to the national level.

Etiological attribution at any level was achieved in only 30.8% of cases, well below the 50–70% identification rates reported in comparable international series (11, 33). Multiple factors are likely to contribute to this diagnostic gap. The widespread availability of over-the-counter antibiotics in Honduras may reduce culture sensitivity upon hospital admission; advanced molecular diagnostic techniques such as 16S rRNA sequencing or next-generation metagenomic sequencing are not routinely available at the institution; sample handling is often suboptimal; and fastidious or slow-growing organisms are intrinsically difficult to culture. In our cohort, the absence of surgical sampling meant that microbiological identification depended on cerebrospinal fluid, blood culture, or serology, none of which has the diagnostic yield of brain tissue analysis. Even when surgical sampling is performed, brain abscess cultures have variable sensitivity (33); the absence of brain tissue in our cohort therefore compounded an already constrained diagnostic context. The confirmed isolation of *Burkholderia pseudomallei* in one case is clinically important, as this pathogen is endemic in tropical environments and may be underrecognized in Central America (12, 25). The polymicrobial etiological pattern in Case 1, identified through cerebrospinal fluid culture and staining in a severely immunocompromised host with cancer, reflects the expected profile of opportunistic infections in this population. Frequent prehospital antibiotic use complicates the selection of empirical therapy and may increase the risk of treatment failure, reinforcing the importance of rational antibiotic policies and structured multidisciplinary protocols in resource-limited settings.

### 4.4 Surgical management

A striking feature of this cohort was the universal absence of therapeutic neurosurgical intervention. Contemporary international series report substantially higher rates of surgical drainage: Tabanlı reported 100% surgical management in 69 adults from Türkiye (35), and the 2024 ESCMID guidelines recommend stereotactic aspiration for abscesses exceeding approximately 2.5 cm in maximal diameter, particularly when accessible (8). In our series, the conservative strategy was clinically justified on a case-by-case basis: deep-seated lesions with concurrent severe immunocompromise, small abscess volumes amenable to medical management, multiple bilateral distributions that limited a meaningful surgical target, and preserved neurological status. Institutional factors also contributed, including limited and variable availability of stereotactic neurosurgical capacity and intraoperative imaging at the institution during the study period. The 0% surgical rate, while defensible for individual patients, departs from international practice and reflects a structural feature of brain abscess management at this referral center. Recognizing this gap is the first step toward establishing protocolized, multidisciplinary management pathways that explicitly weigh surgical drainage when imaging characteristics warrant it (35).

### 4.5 In-hospital outcomes

In-hospital mortality in this series was 0 of 13, which contrasts with figures of 10 to 20% reported in adult cohorts from high-income settings, including the Brouwer meta-analysis (11), the Bodilsen Danish nationwide cohort (4), and the Tabanlı series, which reported 20% mortality (35). Several factors plausibly contributed to the favorable survival profile observed at our center: a preserved Glasgow Coma Scale at admission in 84.6% of patients, a young median age driven by the pediatric component of the cohort, prolonged empirical antimicrobial coverage with adequate central nervous system penetration, and rapid escalation to broader-spectrum or pathogen-directed regimens when initial response was suboptimal. Survival, however, did not translate into uniformly favorable functional outcomes. Long-term epilepsy requiring chronic antiepileptic therapy was established in 46.2% of patients, homonymous hemianopia in 15.4%, and cognitive impairment, hearing loss, or hemiparesis in additional patients. Roughly one in four patients was discharged with moderate disability (mRS 3). These figures should be interpreted with caution given the sample size and the absence of long-term follow-up after discharge, and they should not be cited as evidence that surgery can be safely deferred in similar contexts. The high burden of neurological sequelae underscores that successful infection control does not equate to functional recovery in brain abscess.

### 4.6 Antimicrobial management

Empirical antimicrobial regimens in this cohort followed a three-tier pattern that mapped to clinical risk. A ceftriaxone–metronidazole backbone was used in 5 of 13 patients with uncomplicated solitary abscesses; vancomycin was added in another 5 cases with hardware, post-traumatic, or post-surgical features; and a meropenem-based broad-spectrum regimen was used in 3 high-acuity cases involving severe immunocompromise or meningitis-complicated abscesses. These choices align with the 2024 ESCMID empirical recommendations (8). The absence of microbiological identification in 9 of 13 cases meant that empirical regimens were generally continued for the full course, with limited opportunities for de-escalation. Two pathogen-directed transitions were nonetheless required: Case 1, with polymicrobial cryptococcal, staphylococcal, and nocardial infection identified from cerebrospinal fluid, was transitioned to combined antifungal, antinocardial, and antistaphylococcal therapy planned for 6 to 12 months; Case 13, with presumed central nervous system melioidosis, required intensive intravenous meropenem followed by 6 months of oral trimethoprim–sulfamethoxazole eradication, in accordance with international melioidosis guidelines. Recent pharmacokinetic work has emphasized variable cerebrospinal fluid concentrations of vancomycin and meropenem during CNS infection, reinforcing the need for careful agent selection and, where feasible, therapeutic drug monitoring (36, 37). Case-based experience supports broad empirical coverage with timely transition to targeted therapy when an organism is identified (38). The local management pattern observed at Hospital Escuela aligns with these principles, but its translation into protocolized practice will require prospective data on dose adequacy, treatment duration, and functional outcome.

### 4.7 Clinical presentation

The predominance of seizures (46.2%) and headache (46.2%) as presenting symptoms is consistent with reported clinical patterns (2, 8). A recent meta-analysis confirmed that the clinical presentation of brain abscess remains nonspecific and frequently leads to diagnostic delays (32). Preserved neurological status at admission, with Glasgow Coma Scale 13–15 in 84.6% of patients, is a prognostically favorable feature; a GCS above 12 at presentation has been associated with better outcomes (39). In our cohort, the high proportion of patients presenting with preserved consciousness may have contributed to the favorable in-hospital survival profile reported in Table 7.

### 4.8 Comparison with regional data

Compared with the Colombian series by Acosta Pérez et al. (15), which analyzed 43 cases from a high-complexity university hospital, our findings share similar challenges in etiologic diagnosis but differ in the distribution of predisposing conditions. The Colombian series reported higher rates of osteogenic sources (38.6%) and post-surgical cases, whereas TBI was proportionally more prominent in our cohort. A prospective Iranian study analyzing 110 episodes of infectious brain abscess reported that, when combined with diffusion-weighted imaging sequences, MRI demonstrated a sensitivity of 92% and a specificity of 91% (40). Separately, the 2024 ESCMID guidelines strongly recommend MRI with contrast and DWI as the first-line imaging modality for suspected brain abscess (8). Our greater reliance on CT scanning may therefore have limited diagnostic precision, particularly for early cerebritis stages. CT scanning has lower specificity than MRI for distinguishing brain abscess from ring-enhancing neoplastic lesions such as glioblastoma or metastasis (41).

Compared with the Colombian series by Acosta Pérez et al. (15), which analyzed 43 cases from a high-complexity university hospital, our findings share similar challenges in etiologic diagnosis but differ in the distribution of predisposing conditions; the Colombian series reported higher rates of otogenic sources and post-surgical cases, whereas TBI was proportionally more prominent in our cohort. A contemporary adult series by Tabanlı from a single center in Türkiye (*n* = 69, 2013–2023) reported a comparable male predominance (58%) and frontal–parietal–temporal lesion distribution but differed from our findings in three respects relevant to the Honduran context: 100% surgical management, 72% MRI utilization, and 20% in-hospital mortality (35). The contrast reflects, in part, the resource gradient between the two settings. The distribution of lesion location in our cohort, with parietal and occipital lobes each accounting for 30.8% of dominant lesions, should be interpreted with caution given the small sample size; the parieto-occipital predominance does not reach statistical separation from chance distribution and may not be reproducible in larger series. A prospective Iranian study of 110 brain abscess episodes reported that MRI with diffusion-weighted imaging achieved 92% sensitivity and 91% specificity (40), and the 2024 ESCMID guidelines strongly recommend MRI with contrast and DWI as the first-line imaging modality for suspected brain abscess (8). The 38.5% MRI utilization in our cohort is therefore notable: it reflects equipment availability constraints at the institution during the study period, including prolonged service interruptions; prohibitive out-of-pocket costs in a healthcare system without universal coverage; and clinical decision-making that prioritized prompt initiation of empirical antimicrobial therapy when CT findings were already consistent with brain abscess in the appropriate clinical context. In the three cases without contemporary neuroimaging (Cases 5, 8, and 13), diagnosis relied on documented neurosurgical-team clinical assessment supported by cerebrospinal fluid or serological findings. CT scanning has lower specificity than MRI for distinguishing brain abscess from ring-enhancing neoplastic lesions (41), which is one of the contributors to the broad case definition adopted in this study (Methods, Case Identification and Diagnostic Criteria).

### 4.9 Diagnostic challenges in a resource-limited setting

The absence of documented neuroimaging in 23.1% of cases reflects significant resource constraints. The 2024 ESCMID guidelines strongly recommend MRI with contrast and diffusion-weighted imaging (DWI) for suspected brain abscess, reporting a sensitivity of 92% and specificity of 91% (8). In resource-limited settings, access to MRI is limited by equipment availability, prohibitive costs for uninsured patients, extended wait times, and the absence of 24-h coverage.

### 4.10 Implications for clinical practice

Given the nonspecific clinical presentation and the diagnostic challenges illustrated in this cohort, clinicians should maintain a high index of suspicion for brain abscess in patients with focal neurological signs, persistent headache, or new-onset seizures, particularly when predisposing conditions are present. Given the low etiologic identification rate, empirical antibiotic coverage should broadly address streptococci, staphylococci, and anaerobes; the 2024 ESCMID guidelines recommend ceftriaxone plus metronidazole as the backbone regimen, with vancomycin added when methicillin-resistant *Staphylococcus aureus* is suspected (8). Early neurosurgical consultation is essential, as aspiration or excision provides both therapeutic benefit and the best opportunity for microbiological sampling and etiological identification (8). The complete absence of surgical sampling in our cohort, even in cases that would conventionally have qualified for aspiration, points to the need for explicit, locally adapted multidisciplinary protocols that articulate when and how stereotactic procedures should be considered (35).

### 4.11 Strengths, limitations, and potential biases

This study is the first published report on the epidemiology of brain abscesses in Honduras and provides baseline data for future multicenter prospective studies. Strengths include comprehensive case ascertainment over 5 years at the national referral center and transparent reporting of estimate imprecision through confidence intervals.

This study has several limitations. First, the small sample size (*n* = 13) yields wide confidence intervals and precludes formal inferential analysis; all estimates should be interpreted as descriptive of the institutional cohort rather than representative of national patterns. Second, the retrospective single-center design carries inherent risks of information and selection bias; Hospital Escuela is a national referral center, and its case mix reflects referral pathways for pediatric trauma, complex infectious disease, and complicated neurosurgical cases that are not generalizable to other Honduran centers. Third, the case definition was permissive: 10 of 13 cases were identified by ring-enhancing lesions on CT or MRI in the appropriate clinical context, and 3 cases without contemporary neuroimaging were attributed by documented neurosurgical-team clinical assessment supported by cerebrospinal fluid or serological findings. No patient underwent surgical sampling, and intraoperative or histopathological confirmation of brain tissue was therefore not available in any case. Misclassification of competing entities such as tuberculomas, neurocysticercosis, toxoplasmosis, and ring-enhancing neoplastic lesions cannot be formally excluded, and the broad inclusion criteria likely contribute to the low etiologic identification rate. Fourth, contiguous otorhinolaryngological sources (otitis media, sinusitis, mastoiditis) were not systematically documented and may be underrepresented in the available records. Fifth, one record contained an unverifiable entry of alcohol use disorder in a pediatric patient (Case 11) that was excluded from the main predisposing-condition analysis. Sixth, the low etiologic identification rate (30.8%) constrains conclusions about the local microbiological landscape and reflects, in part, the unavailability of molecular diagnostic platforms during the study period. Seventh, magnetic resonance imaging was performed in fewer than half of the patients (5 of 13 by any modality), reflecting equipment availability and prolonged service interruptions at the institution during portions of the study period, prohibitive out-of-pocket costs in a healthcare system without universal coverage, and reliance on alternative diagnostic correlates in selected cases; this further limits diagnostic precision, particularly for early cerebritis. Eighth, the absence of long-term follow-up after discharge prevents assessment of late epilepsy, neurocognitive recovery, antibiotic-related complications, and abscess relapse, all of which are clinically relevant outcomes in brain abscess. Ninth, sub-registration of cases managed at peripheral facilities without referral to Hospital Escuela is an additional source of epidemiological uncertainty that this study cannot quantify.

## 5 Conclusion

This institutional case series represents the first published description of brain abscess epidemiology in Honduras. In this cohort of 13 patients, all abscesses were supratentorial, consistent with global literature. The distributions of sex, age group, and predisposing conditions were heterogeneous and are presented as descriptive observations without formal inferential claims. The high proportion of cases without etiologic identification (69.2%) and the limited availability of advanced neuroimaging underscore critical diagnostic gaps that must be addressed through improved laboratory infrastructure and broadened access to MRI in resource-limited settings.

Future research should prioritize prospective, multicenter studies with larger sample sizes, structured documentation of treatment decisions and longitudinal outcomes, implementation of enhanced microbiological protocols including molecular and metagenomic diagnostics, and follow-up beyond hospital discharge. The development of locally adapted multidisciplinary protocols, articulated by neurology, neurosurgery, infectious diseases, and microbiology services, is a particularly pressing need in Central America. This study contributes a first institutional clinical-epidemiological characterization of brain abscess in Honduras and provides baseline data on demographics, neuroanatomical distribution, predisposing conditions, microbiological yield, treatment patterns, and short-term outcomes that can guide future prospective research in the region.

## Data Availability

The original contributions presented in the study are included in the article/Supplementary material, further inquiries can be directed to the corresponding author.
