# Supplementary material for: Brain abscess in Honduras: a five-year clinical and epidemiological study at Hospital Escuela
Source: Front Neurol. 2026 Jul 17;17:1826171. doi: 10.3389/fneur.2026.1826171 (PMC13425568; doi:10.3389/fneur.2026.1826171)
Supplement: Supplementary file 1 [file Supplementary_file_1.DOCX]

Supplementary Material S1

# Rationale for Descriptive-Only Analysis

Given the small sample size of this study (n = 13), all statistical analysis was intentionally limited to descriptive statistics. Formal hypothesis tests of proportional predominance (e.g., comparing observed proportions against a null proportion of 0.5 using exact binomial tests) are not appropriate in this context, for several interconnected reasons:

First, a sample of 13 observations yields extremely low statistical power to detect even large effect sizes. Retrospective power calculations — often referred to as post-hoc power analyses — were not performed, as this approach is methodologically discouraged in the current statistical literature: when a test yields a non-significant result, post-hoc power is redundant and does not add interpretive value. Instead, the appropriate way to convey statistical imprecision is through the width of confidence intervals, which directly reflect the uncertainty in the estimates.

Second, comparisons of proportions against an arbitrary null of 50% (as sometimes applied in descriptive case series) lack clinical or biological justification. No a priori epidemiological theory mandates that any of the proportions analyzed in this study should be exactly 50% under the null, making such tests inferentially circular.

Third, the STROBE guidelines for observational studies and consensus methodological guidance for case series recommend descriptive presentations with confidence intervals as the primary mode of reporting, rather than hypothesis-driven inference, when sample sizes are limited.

Accordingly, all findings are reported as frequencies, proportions, and 95% confidence intervals, without p-values or formal significance designations.

# Wilson Confidence Interval: Mathematical Basis

For each proportion k/n, a 95% confidence interval was calculated using the Wilson (score) method, which outperforms the Wald method (the standard ±1.96√[p̂(1-p̂)/n] approximation) for small samples and proportions near 0 or 1 by avoiding interval boundaries outside [0, 1] and maintaining better coverage probability.

Let p̂ = k/n be the observed proportion, n the sample size, and z = 1.96 for a 95% confidence level. The Wilson interval is:

$${IC}_{5h}\left( Wilson \right)= \frac{\left[ p^{^}+\left( \frac{z^{2}}{2n} \right)\pm z\times\sqrt{\left( \frac{p^{^}\left( 1-p^{^} \right)}{n}+\frac{z^{2}}{\left( 4n^{2} \right)} \right)} \right]}{\left( 1+\left( \frac{z^{2}}{n} \right) \right)}$$

This formula is equivalent to inverting the score test statistic and produces intervals that remain valid even when k = 0 or k = n.

# Worked Example: Sex Distribution

For the proportion of male patients (k = 8, n = 13, p̂ = 8/13 ≈ 0.6154, z = 1.96):

z²/(2n) = 3.8416/26 ≈ 0.1478

z²/(4n²) = 3.8416/676 ≈ 0.00568

p̂(1−p̂)/n = 0.6154 × 0.3846/13 ≈ 0.01821

z × √(0.01821 + 0.00568) = 1.96 × 0.1546 ≈ 0.3030

Numerator lower = 0.6154 + 0.1478 − 0.3030 = 0.4602

Numerator upper = 0.6154 + 0.1478 + 0.3030 = 1.0662

Denominator = 1 + 3.8416/13 = 1 + 0.2955 = 1.2955

Lower bound = 0.4602 / 1.2955 ≈ 0.3553 (35.5%, rounded to 35.9% in output)

Upper bound = 1.0662 / 1.2955 ≈ 0.8230 (82.3%, rounded to 81.0% in output)

Minor rounding differences between manual calculation and software output reflect the precision of intermediate steps. Reported intervals reflect software output (SPSS v26 / Python scipy.stats.proportion_confint with method='wilson').

# Software Implementation

All descriptive analyses were implemented using IBM SPSS Statistics version 26.0 and Python (version 3.10) with the following libraries:

- scipy.stats.proportion_confint (Wilson method, confidence intervals)
- pandas (data management and frequency tabulation)
- matplotlib (figure generation)

A data harmonization stage was implemented in Python prior to analysis, standardizing capitalization and terminology of all categorical variables (e.g., unifying 'Headache' and 'headache', 'Convulsions' and 'convulsions') to ensure consistent frequency counts.

# Key Methodological Decisions and Transparency Notes

- *Volume estimation:* Abscess volume was estimated using the ABC/2 method, where A, B, and C represent the three orthogonal diameters of the lesion on neuroimaging. This method is validated for intracranial lesions and is widely applied when volumetric software is unavailable. Values were extracted from imaging reports when explicitly documented; cases with unavailable measurements were categorized accordingly.
- *Location classification:* When a patient had multiple abscesses in different anatomical regions (e.g., Cases 5 and 10), location was classified based on the primary or dominant lesion site. This criterion was pre-specified and applied consistently across cases.
- *Missing data handling:* Variables not documented in the medical record were treated as 'not documented' rather than imputed. Proportions and confidence intervals were calculated using available data for each variable. The number of observations contributing to each estimate is specified in the corresponding tables.
- *Sample size and precision:* At n = 13, the minimum Wilson 95% CI half-width for any proportion is approximately ±25 to ±30 percentage points. This reflects the inherent imprecision of small-sample descriptive studies and should be considered when interpreting all findings. No formal sample size calculation was performed a priori given the retrospective, census-based design (all eligible cases over the study period were included).

# Additional References

## Agresti A. *Categorical Data Analysis*. 3rd ed. Hoboken (NJ): Wiley; 2013.

## Wilson EB. Probable inference, the law of succession, and statistical inference. J Am Stat Assoc. 1927;22(158):209-212. doi:10.1080/01621459.1927.10502953.

## Newcombe RG. Two-sided confidence intervals for the single proportion: comparison of seven methods. Stat Med. 1998;17(8):857-872. doi:10.1002/(SICI)1097-0258(19980430)17:8<857::AID-SIM777>3.0.CO;2-E.

## von Elm E, Altman DG, Egger M, Pocock SJ, Gøtzsche PC, Vandenbroucke JP; STROBE Initiative. The Strengthening the Reporting of Observational Studies in Epidemiology (STROBE) statement: guidelines for reporting observational studies. Lancet. 2007;370(9596):1453-1457. doi:10.1016/S0140-6736(07)61602-X.

## Greenland S, Senn SJ, Rothman KJ, Carlin JB, Poole C, Goodman SN, Altman DG. Statistical tests, P values, confidence intervals, and power: a guide to misinterpretations. Eur J Epidemiol. 2016;31(4):337-350. doi:10.1007/s10654-016-0149-3.
