# Supplementary material for: Brain abscess in Honduras: a five-year clinical and epidemiological study at Hospital Escuela
Source: Front Neurol. 2026 Jul 17;17:1826171. doi: 10.3389/fneur.2026.1826171 (PMC13425568; doi:10.3389/fneur.2026.1826171)
Supplement: Supplementary file 2 [file Supplementary_file_2.DOCX]

Supplementary Material S2

**STROBE Checklist for Observational Studies — Case Series**

This checklist documents the application of the STROBE (Strengthening the Reporting of Observational Studies in Epidemiology) guidelines to the present manuscript. STROBE was originally developed for cohort, case-control, and cross-sectional studies; items have been adapted here to a retrospective descriptive case series following the conventions established in the methodological literature [von Elm E, et al. Lancet 2007; 370:1453–1457]. Items not applicable to this design are explicitly noted.

Each item is rated as: “Yes” (fully addressed), “Partial” (addressed with acknowledged limitations), or “N/A” (not applicable to a retrospective descriptive case series).

| **Item** | **STROBE Recommendation** | **Applicable** | **Location in Manuscript** | **Section / Page** |
| --- | --- | --- | --- | --- |
| **TITLE AND ABSTRACT** | | | | |
| **1(a)** | Indicate the study design with a commonly used term in the title or abstract. | **Yes** | Abstract (Methods) | Abstract, line 4 |
| **1(b)** | Provide in the abstract an informative and balanced summary of what was done and what was found. | **Yes** | Abstract | Abstract (all sections) |
| **INTRODUCTION** | | | | |
| **2** | Explain the scientific background and rationale for the investigation being reported. | **Yes** | Section 1 | §1. Introduction, ¶1–4 |
| **3** | State specific objectives, including any prespecified hypotheses. | **Yes** | Section 1 | §1. Introduction, final ¶ |
| **METHODS** | | | | |
| **4** | Present key elements of study design early in the paper. | **Yes** | Section 2.1 | §2.1. Study Design and Setting |
| **5** | Describe the setting, locations, and relevant dates, including periods of recruitment, exposure, follow-up, and data collection. | **Yes** | Section 2.1 | §2.1: Hospital Escuela, Tegucigalpa; Jan 2018–Dec 2023 |
| **6** | Case series: Give the eligibility criteria, and the sources and methods of case ascertainment and selection. | **Yes** | Section 2.2 | §2.2. Case Identification and Diagnostic Criteria |
| **7** | Clearly define all outcomes, exposures, predictors, potential confounders, and effect modifiers. Give diagnostic criteria, if applicable. | **Yes** | Section 2.3 | §2.3. Variables and Data Collection; diagnostic criteria in §2.2 |
| **8** | For each variable of interest, give sources of data and details of methods of assessment (measurement). Describe comparability of assessment methods if there is more than one group. | **Yes** | Section 2.3 | §2.3: ICD-10 codes, medical records, standardized extraction instrument, ABC/2 volume method |
| **9** | Describe any efforts to address potential sources of bias. | **Yes** | Section 4.8 | §4.8. Strengths, Limitations, and Potential Biases |
| **10** | Explain how the study size was arrived at. | Partial | Section 2.1 | §2.1: total universe of cases (n = 13) over the 5-year period; no a priori sample size calculation performed (acknowledged as limitation) |
| **11** | Explain how quantitative variables were handled in the analyses. If applicable, describe which groupings were chosen and why. | **Yes** | Section 2.3 | §2.3: age groups (≤15, 16–59, ≥60 years); volume categories (<1, 1–10, >10 cm³) |
| **12(a)** | Describe all statistical methods, including those used to control for confounding. | **Yes** | Section 2.4 | §2.4. Statistical Analysis: frequencies, proportions, Wilson 95% CI |
| **12(b)** | Describe any methods used to examine subgroups and interactions. | N/A | — | Not applicable (descriptive case series; no subgroup analysis) |
| **12(c)** | Explain how missing data were addressed. | **Yes** | Sections 2.3, 4.8 | §2.3: data harmonization note; §4.8: missing imaging, incomplete microbiological documentation acknowledged as limitations |
| **12(d)** | If applicable, explain how loss to follow-up was addressed. | N/A | — | Not applicable (retrospective case series; no prospective follow-up) |
| **12(e)** | Describe any sensitivity analyses. | N/A | — | Not applicable (sample size precludes sensitivity analyses) |
| **RESULTS** | | | | |
| **13(a)** | Report the numbers of individuals at each stage of the study. | **Yes** | Section 3.1 | §3.1: 13 records identified, all included |
| **13(b)** | Give reasons for non-participation at each stage. | **Yes** | Section 2.2 | §2.2: exclusion criteria stated (incomplete records, outside study period, reclassified lesions) |
| **13(c)** | Consider use of a flow diagram. | Partial | — | Flow diagram not included; case ascertainment described narratively in §2.2. Recommended for future submissions. |
| **14(a)** | Give characteristics of study participants and information on exposures and potential confounders. | **Yes** | Tables 1–5; Table S1 | §3.1–3.5; Tables 1–5; Supplementary Table S1 |
| **14(b)** | Indicate the number of participants with missing data for each variable of interest. | **Yes** | Tables 4–6; §4.8 | Missing neuroimaging: 3/13 (Table 5); missing microbiological data: 9/13 (Table 6); CRP not analyzable (§3.6) |
| **15** | Report numbers of outcome events or summary measures over time. | Partial | Sections 3.1–3.6 | §3.1–3.6: frequencies and proportions with 95% CI reported for all variables. In-hospital outcomes not systematically documented (acknowledged in §4.8) |
| **16(a)** | Give unadjusted estimates and, if applicable, confounder-adjusted estimates and their precision. | **Yes** | Tables 1–6; Table S2 | 95% Wilson CI reported for all proportions. No adjustment performed (descriptive study) |
| **16(b)** | Report category boundaries when continuous variables were categorized. | **Yes** | Section 2.3 | §2.3: age group cut-offs; volume categories defined |
| **16(c)** | If relevant, consider translating estimates of relative risk into absolute risk for a meaningful time period. | N/A | — | Not applicable (descriptive case series; no relative risk estimates) |
| **17** | Report other analyses done — e.g., analyses of subgroups and interactions, and sensitivity analyses. | N/A | — | No additional analyses performed. Wilson CI calculations detailed in Supplementary Material S1 |
| **DISCUSSION** | | | | |
| **18** | Summarise key results with reference to study objectives. | **Yes** | Section 5 | §5. Conclusions |
| **19** | Discuss limitations of the study, taking into account sources of potential bias or imprecision. | **Yes** | Section 4.8 | §4.8: small sample, retrospective design, selection bias, low microbiological yield, missing ORL documentation, incomplete outcomes |
| **20** | Give a cautious overall interpretation of results considering objectives, limitations, multiplicity of analyses, results from similar studies, and other relevant evidence. | **Yes** | Sections 4.1–4.8 | §4.1–4.8: contextualization with international literature; cautious language throughout |
| **21** | Discuss the generalisability (external validity) of the study results. | **Yes** | Sections 4.8, 5 | §4.8: single-center, tertiary referral; §5: results described as institutional observations not generalizable to national level |
| **OTHER INFORMATION** | | | | |
| **22** | Give the source of funding and the role of the funders for the present study and, if applicable, for the original study on which the present article is based. | **Yes** | Funding section | Funding section: no external funding received |

**Legend:**

| **Yes** | Item fully addressed in the manuscript. |
| --- | --- |
| **Partial** | Item partially addressed; limitations acknowledged in §4.8. |
| N/A | Not applicable to a retrospective descriptive case series. |

Reference: von Elm E, Altman DG, Egger M, Pocock SJ, Gøtzsche PC, Vandenbroucke JP; STROBE Initiative. Strengthening the Reporting of Observational Studies in Epidemiology (STROBE) statement: guidelines for reporting observational studies. Lancet 2007; 370:1453–1457. https://doi.org/10.1016/S0140-6736(07)61602-X

The full STROBE checklist and explanatory document are available at: https://www.strobe-statement.org
