# Supplementary material for: Brain abscess in Honduras: a five-year clinical and epidemiological study at Hospital Escuela
Source: Front Neurol. 2026 Jul 17;17:1826171. doi: 10.3389/fneur.2026.1826171 (PMC13425568; doi:10.3389/fneur.2026.1826171)
Supplement: Supplementary file 3 [file Supplementary_file_3.DOCX]

Supplementary Material Figures


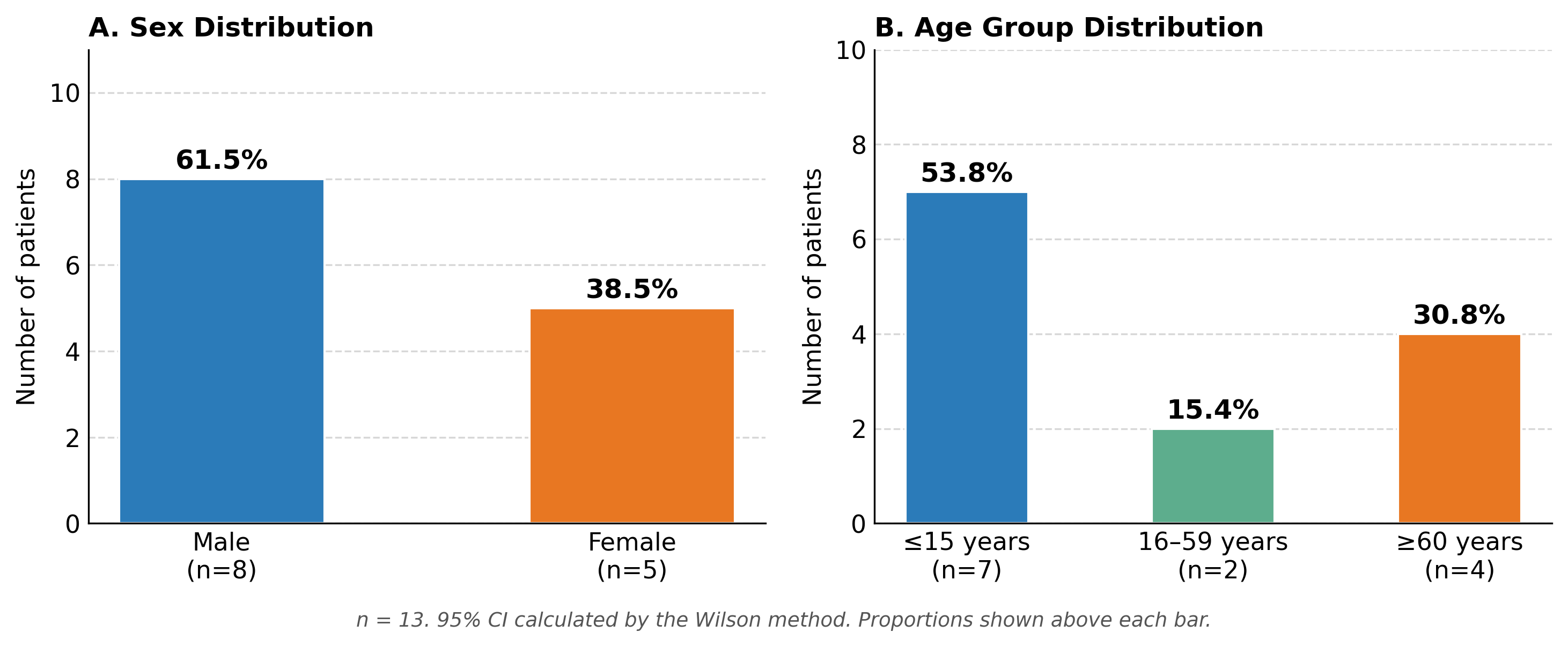


**Figure S1: Demographic distribution of brain abscess patients (n = 13).** (A) Distribution by sex. Males represented 61.5% (n = 8) and females 38.5% (n = 5). (B) Distribution by age group: pediatric (≤15 years) 53.8% (n = 7), adult 16–59 years 15.4% (n = 2), elderly (≥60 years) 30.8% (n = 4). Proportions shown above each bar. 95% confidence intervals (Wilson method) are reported in Table 1 and Table S2. No formal tests of predominance were applied given the small sample size.


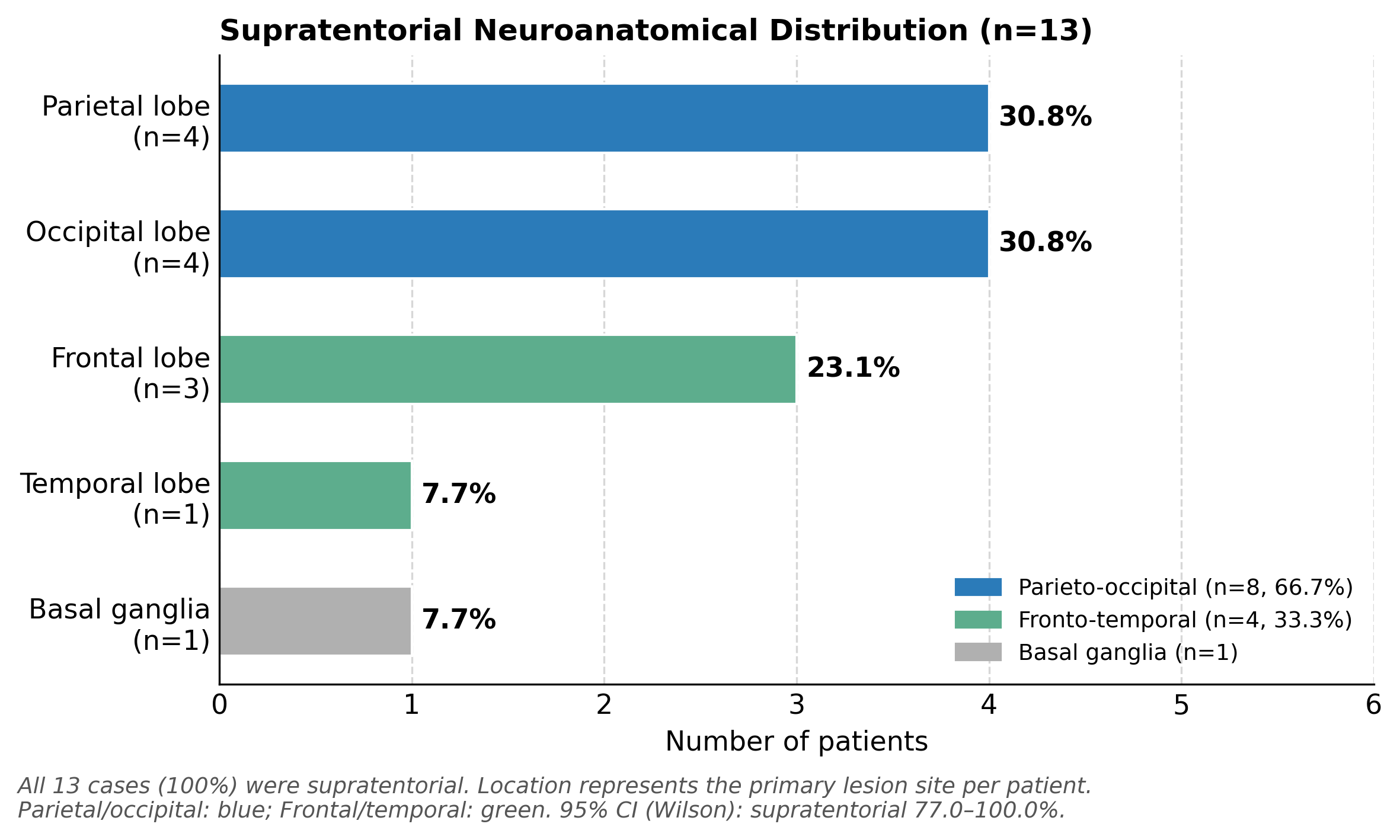


**Figure S2: Neuroanatomical distribution of brain abscesses (n = 13).** Distribution of the dominant lesion site per patient. All 13 cases (100%; 95% CI: 77.0–100.0%) were supratentorial. Parietal and occipital lobes each accounted for 30.8% (n = 4), followed by frontal lobe (23.1%; n = 3), temporal lobe (7.7%; n = 1), and basal ganglia (7.7%; n = 1). When two lobes were simultaneously involved, the dominant lesion determined primary classification. Parieto-occipital territory (blue): 61.5%; fronto-temporal territory (green): 30.8%. See Table S5 for neuroimaging details.


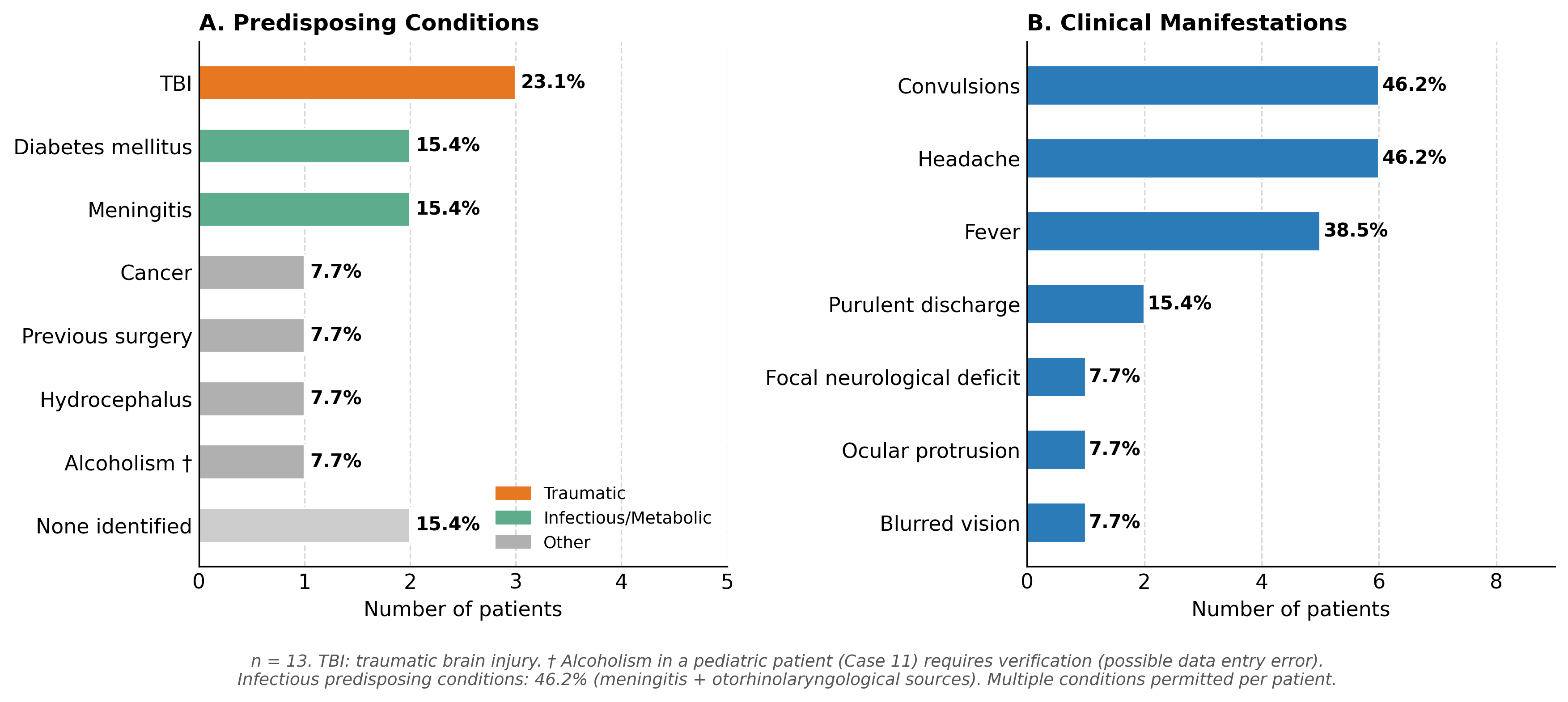


**Figure S3: Predisposing conditions (A) and clinical manifestations (B) in brain abscess patients (n = 13).** (A) Predisposing conditions. Traumatic brain injury (TBI) was the most frequent individual condition (23.1%; n = 3), followed by diabetes mellitus and meningitis (15.4% each). Contiguous otorhinolaryngological sources (otitis media, sinusitis, mastoiditis) were not systematically documented in the available records and may be underrepresented. One record with an unverifiable entry of alcohol use disorder in a pediatric patient (Case 11) is excluded from this figure and retained in Table S1 with an explanatory footnote. Multiple predisposing conditions permitted per patient. See Table S8 for full categorization. (B) Clinical manifestations at presentation. Seizure and headache were most frequent (46.2% each), followed by fever (38.5%). The classic triad of headache, fever, and focal neurological deficit was documented in at most two patients. See Table S3 for complete frequency analysis.


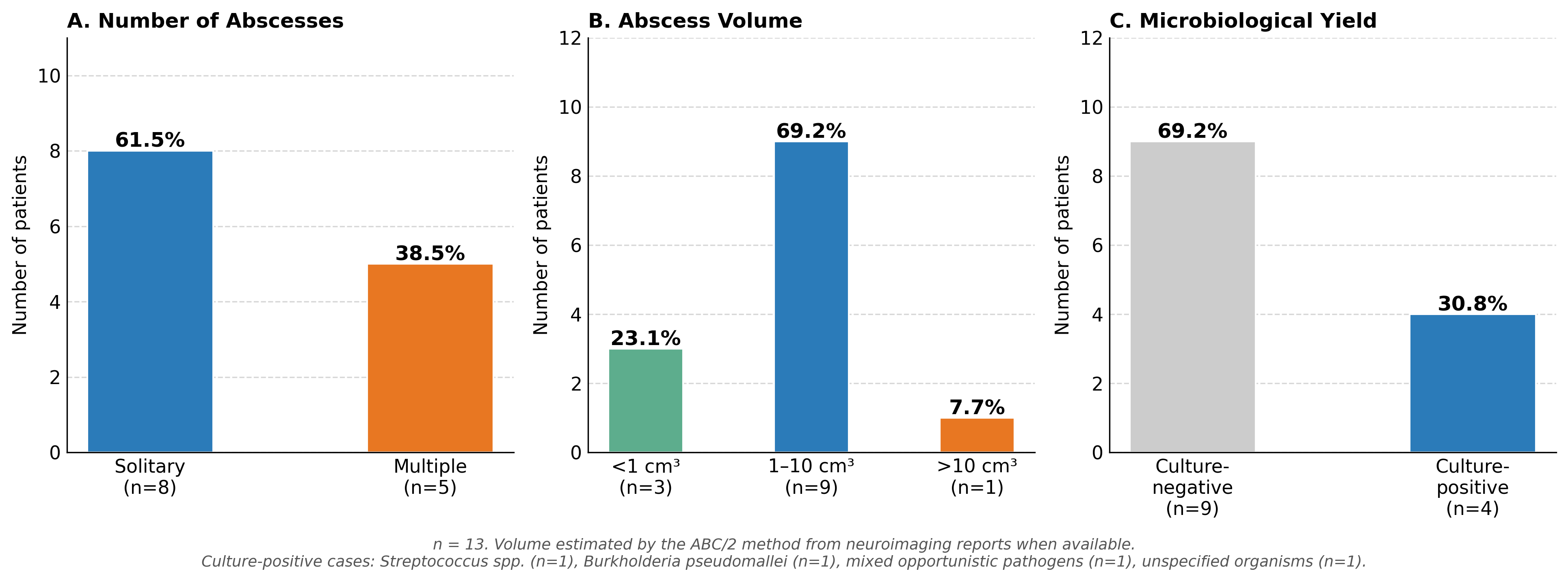


**Figure S4: Abscess characteristics (A, B) and etiologic identification yield (C) (n = 13).** (A) Number of abscesses per patient: solitary 61.5% (n = 8), multiple 38.5% (n = 5). (B) Estimated abscess volume by the ABC/2 method: predominantly medium-sized (1–10 cm³; 69.2%; n = 9). (C) Etiologic identification yield. Etiologic attribution of any level was achieved in 30.8% of cases (4/13), whereas direct microbiological confirmation from CSF was available in only one case; 69.2% remained without etiologic identification. Three levels of evidence were documented: (1) culture-confirmed with histopathological corroboration (Cryptococcus neoformans / Staphylococcus spp. / Nocardia spp.; one case); (2) serology/CSF-supported (Streptococcus spp. and Burkholderia pseudomallei; two cases); (3) indirect CSF evidence only (one case). See Table S6 for three-category classification.
